# Supplementary material for: A Diagnostic Strategy for Gauging Individual Humoral Ex Vivo Immune Responsiveness Following COVID-19 Vaccination
Source: Vaccines (Basel). 2022 Jun 29;10(7):1044. doi: 10.3390/vaccines10071044 (PMC9322304; doi:10.3390/vaccines10071044)
Supplement: Supplementary file 1 [file vaccines-10-01044-s001.zip › vaccines-1771866-supplementary.pdf]

Data Supplement

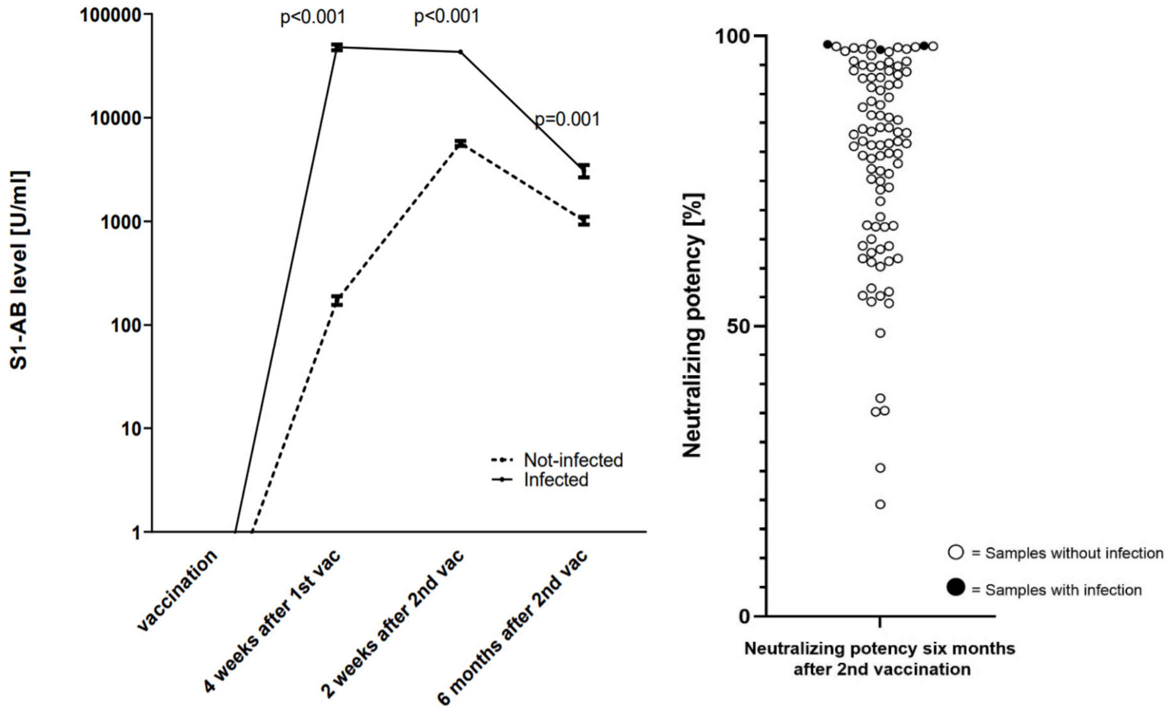

(**A**) (**B**)

Supplementary Figure S1. Impact of prior SARS-CoV-2 infection on vaccination-induced Antibodies against the SARS-CoV-2 spike (S1) protein receptor binding domain (RBD) (S1-AB) serum levels: (**A**) Mean values ± SEM of S1-AB levels measured four weeks after first, two weeks after second and six months after second vaccination. Post-infection and non-infected samples indicated by open and closed symbols, respectively. (**B**) Neutralizing potency six months after second vaccination measured by NeutraLISA. Brackets indicate significant ($p < 0.001$) difference of the mean. Closed symbols: SARS-CoV-2 infection prior to vaccination. Numbers of included participants see Supplementary Table S1.

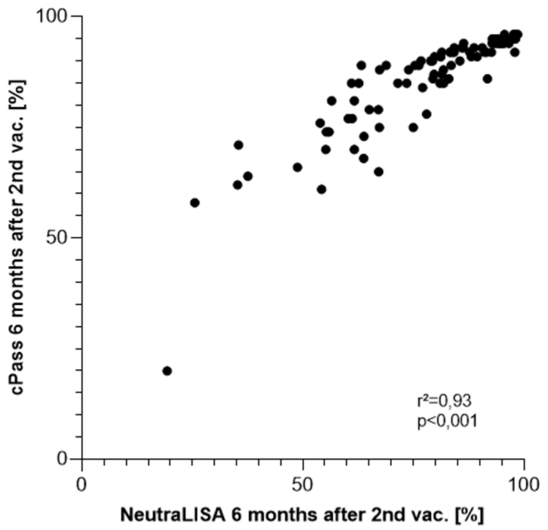

Supplementary Figure S2. Correlation of NeutraLISA and cPass six months after second vaccination: (r = 0,932, $p < 0.001$). Numbers of included participants see Supplementary Table S1.

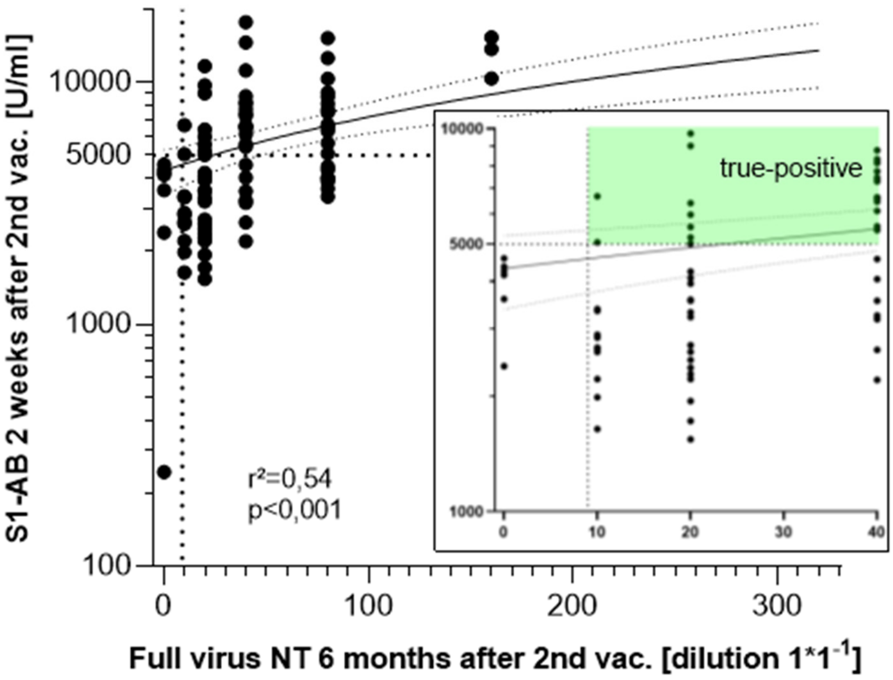

Supplementary Figure S3. Levels of Antibodies against the SARS-CoV-2 spike (S1) protein receptor binding domain (RBD) (S1-AB) and full virus neutralization test (full virus NT) measured two weeks after second vaccination: Linear regression of the data and 95%-confidence interval indicated by solid and dashed lines, respectively (r^2 = 0.54, p < 0.001), insert: blow-up of low-level range. Numbers of included participants see Supplementary Table S1.

Supplementary Table S1. Numbers of participants included in each analysis of the study.

Test	Timepoint	n
S1-AB	48 hours befor first vaccination	3 *
	Four weeks after first vaccination	113
	Two weeks after second vaccination	116
	Six months after second vaccination	95
N-AB	48 hours befor first vaccination	124
	Four weeks after first vaccination	113
	Two weeks after second vaccination	116
	Six months after second vaccination	95
NeutraLISA	Four weeks after first vaccination	113
	Two weeks after second vaccination	116
	Six months after second vaccination	95
cPass	Four weeks after first vaccination	113
	Two weeks after second vaccination	116
	Six months after second vaccination	95
Full virus NT	Six months after second vaccination	89

* Only determined for N-AB-positive participants.
